# Endoscopic vacuum therapy successfully treats postoperative esophageal rupture after laparoscopic-assisted total gastrectomy: a full therapeutic course report

**DOI:** 10.1055/a-2836-1575

**Published:** 2026-04-15

**Authors:** Jiaming Liao, Ruiqi Wang, Qisen Zhu, Min Li, Yunhong Tian

**Affiliations:** 1 662481 Department of General Surgery, Nanchong Central Hospital Affiliated to North Sichuan Medical College, Nanchong, China; 2 Department of General Surgery, Beijing Anzhen Nanchong Hospital of Capital Medical University and Nanchong Central Hospital, Nanchong, China

Esophageal rupture is a rare but life-threatening postoperative complication of gastric cancer surgery associated with substantial morbidity and mortality if not treated promptly and appropriately 1 2 . Traditional treatments, including surgical repair and stenting, are associated with several limitations 3 . Endoscopic vacuum therapy (EVT), as a minimally invasive alternative, may offer advantages in promoting wound healing and improving clinical outcomes for esophageal leakage 4 . Although EVT has been reported for gastrointestinal defects, previously published cases have either involved small esophageal leaks or lacked documentation of a complete therapeutic course with a video. Here, we present a video case report demonstrating the successful management of a large esophageal rupture following the gastric cancer surgery using EVT.

**Fig. 1:**
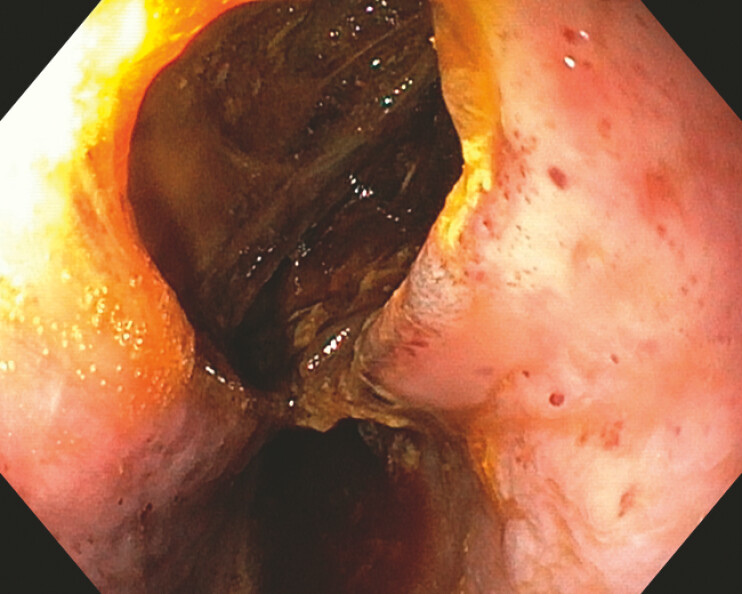
Endoscopy revealed a large longitudinal esophageal rupture measuring approximately 3–4 cm in length, accompanied by a mediastinal abscess measuring 6 × 7 cm.

A 75-year-old man with gastric cancer underwent laparoscopic-assisted total gastrectomy with esophagojejunostomy. The postoperative course was uneventful. On postoperative day 27, the patient developed severe chest pain following postprandial vomiting. Endoscopy revealed a large longitudinal esophageal rupture measuring approximately 3–4 cm in length, located 30 cm from the incisors, and complicated by a mediastinal abscess cavity measuring 6 cm × 7 cm ( Fig. 1 ). Seven EVT sessions were performed at 5- to 7-day intervals ( Video 1 ). The patient was managed on a regular ward and was not admitted to the intensive care unit. Each EVT placement was performed under general anesthesia without endotracheal intubation, and each procedure lasted approximately 20–30 minutes.

Endoscopic vacuum therapy in the management of esophageal rupture.Video 1

The EVT system was assembled as follows. First, open-cell polyurethane foam (sponge) was manually trimmed to match the size of the defect. The sponge was shaped into a cylinder measuring approximately 6 cm in length and 2 cm in diameter and was then secured to the tip of a 6.0 Fr/18 silicone tube using sutures. The other end of the tube was connected to the hospital central vacuum system. This assembly technique is similar to that described in previous endoscopic studies 5 . Continuous negative pressure (−100 to −125 mmHg) was applied.

**Fig. 2:**
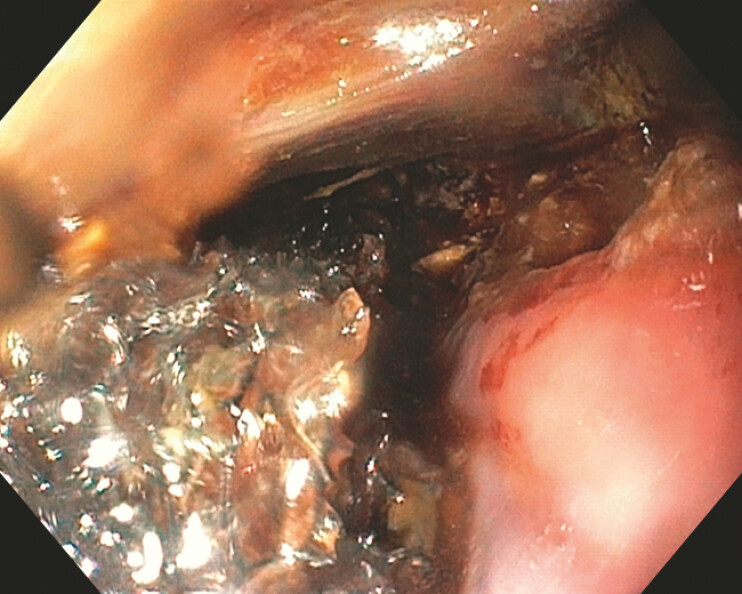
The EVT device was placed into the mediastinal abscess cavity. EVT, endoscopic vacuum therapy.

The sponge was placed under endoscopic guidance. During the first three of a total of seven EVT sessions, the sponge was positioned directly within the defect ( Fig. 2 ). As the defect gradually decreased in size, the sponge volume was slightly reduced in subsequent sessions. In the later sessions, the sponge was placed intraluminally within the esophagus.

**Fig. 3:**
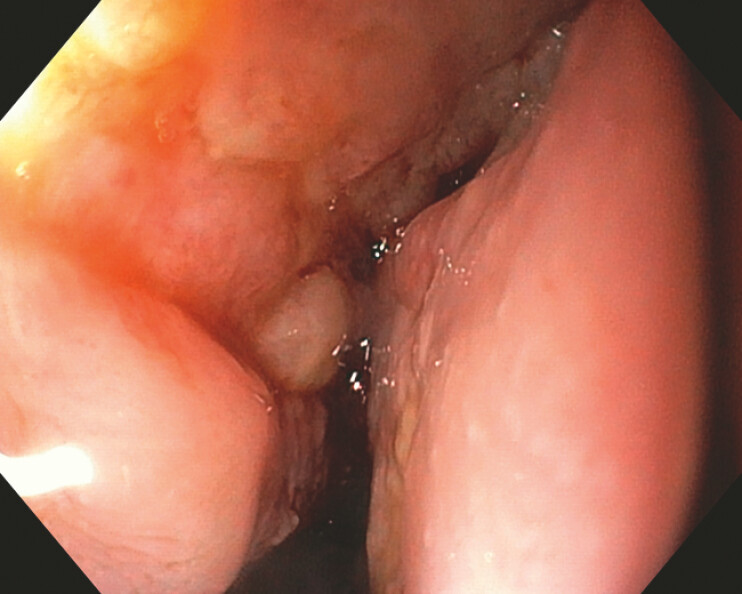
Granulation tissue and purulent coating at the rupture site.

**Fig. 4:**
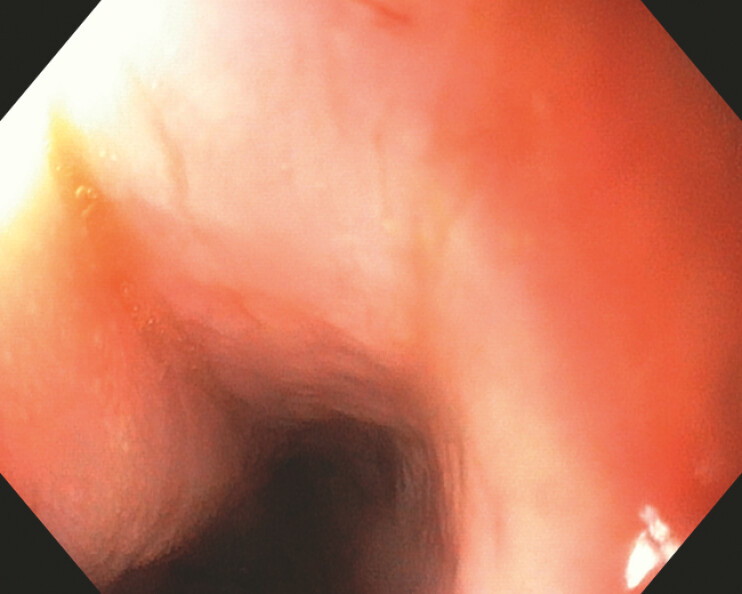
Endoscopy confirmed complete healing of the esophageal rupture.

**Fig. 5:**
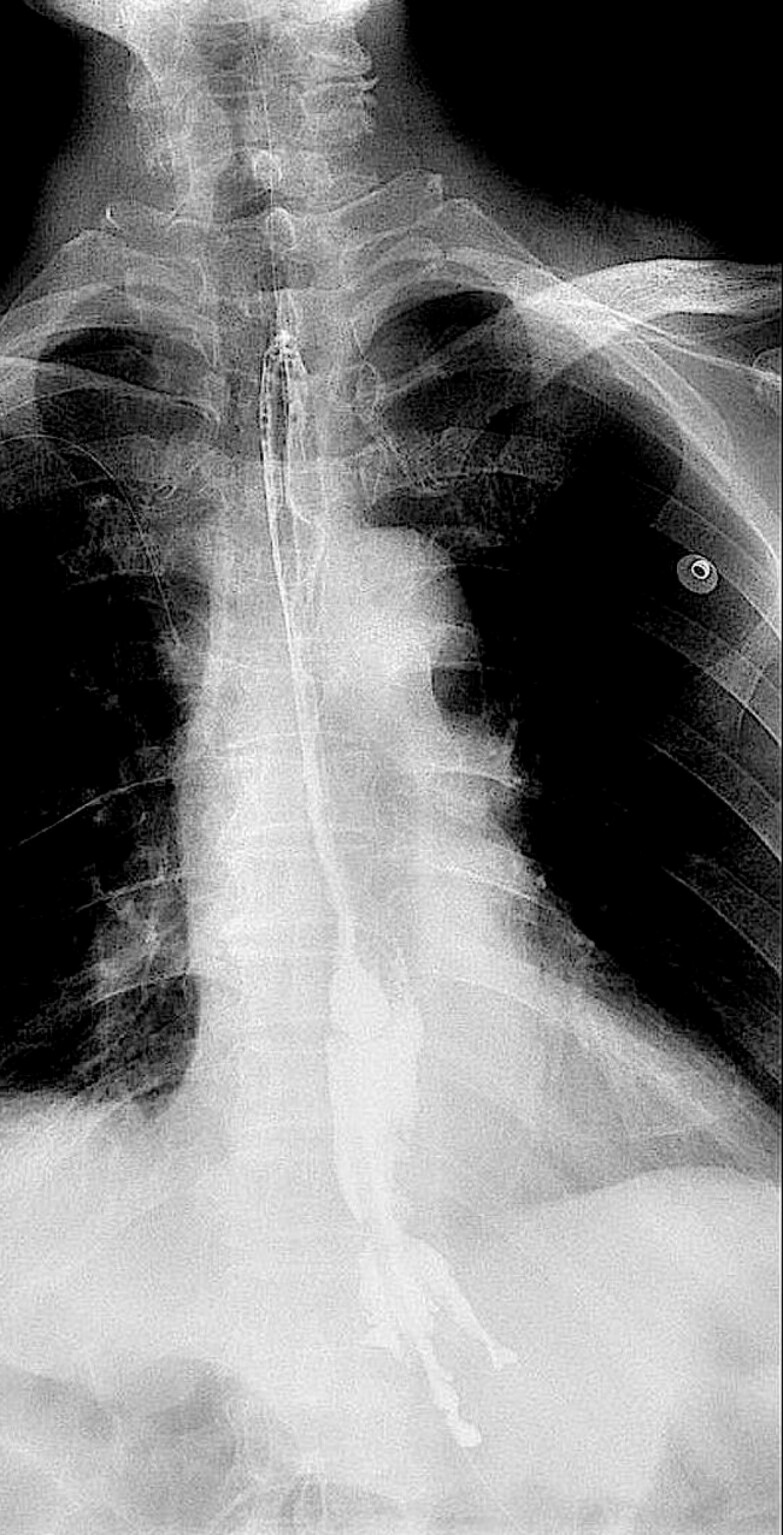
Postoperative upper gastrointestinal radiography.

On day 14 after the initial EVT, the esophageal rupture site demonstrated newly formed granulation tissue ( Fig. 3 ). After 28 days of EVT treatment, the size of the defect was reduced with continued granulation tissue proliferation. At the 1-week follow-up examination after completion of the full therapeutic course, follow-up endoscopy confirmed the complete closure of the esophageal rupture ( Fig. 4 ). An upper gastrointestinal contrast study demonstrated smooth passage of contrast through the esophagus without evidence of leakage or stenosis, confirming successful healing ( Fig. 5 ).

To our knowledge, this is the first video report documenting the complete EVT treatment course for a large esophageal rupture. The present case suggests that EVT may represent a safe and effective minimally invasive treatment option and provides clinical insights into its potential use in large esophageal perforations, including Boerhaave syndrome.

Endoscopy_UCTN_Code_TTT_1AO_2AI
